# Hypercalcemia worsened after vitamin D supplementation in a sarcoidosis patient: A case report

**DOI:** 10.1097/MD.0000000000030883

**Published:** 2022-10-07

**Authors:** Kimito Mio, Kotaro Haruhara, Akihiro Shimizu, Kentaro Oshiro, Rena Kawai, Masato Ikeda, Takashi Yokoo

**Affiliations:** a Division of Nephrology and Hypertension, Department of Internal Medicine, The Jikei University School of Medicine, Tokyo, Japan.

**Keywords:** corticosteroids, hypercalcemia, granulomatous disease, vitamin D supplementation

## Abstract

There are many causes of hypercalcemia, with hyperparathyroidism and malignancy accounting for 90% of cases. Sarcoidosis and the intake of vitamin D supplements may also cause hypercalcemia, although the occurrence rate is low if only one is involved. We herein report a sarcoidosis patient who developed hypercalcemia after taking cholecalciferol (vitamin D supplement) for a year.

**Patient concern:**

A 62-year-old Japanese man presented with hypercalcemia and acute kidney injury along with symptoms of fatigue and appetite loss while being followed up for sarcoidosis.

**Diagnoses:**

We determined that a combination of cholecalciferol supplementation and sarcoidosis had led to hypercalcemia for several reasons. First, hypercalcemia had not been noted when this patient had first been admitted due to sarcoidosis-related respiratory failure several years earlier, which we presumed that was the highest sarcoidosis disease activity. Second, low serum 25-OH Vit.D_3_ and high 1,25-(OH)_2_ Vit.D_3_ levels were noted despite cholecalciferol supplementation for a year, suggesting that 1-α-hydroxylase overexpression caused by sarcoidosis accelerated the conversion from 25-OH Vit.D_3_ to 1,25-(OH)_2_ Vit.D_3_.

**Interventions:**

Although initially resistant to preservative management, the hypercalcemia promptly improved after starting corticosteroid treatment.

**Outcomes:**

Hypercalcemia and acute kidney injury were normalized after corticosteroid treatment.

**Lessons:**

We should be aware of patients’ medications, especially in patients with granulomatosis disease. The concomitant measurement of 25-OH Vit.D_3_ and 1,25-(OH)_2_ Vit.D_3_ levels is useful for determining the cause of hypercalcemia.

## 1. Introduction

Hypercalcemia is an emergent status often encountered by primary care physicians. The symptoms of hypercalcemia are nonspecific and depend on the severity and rapidity of its occurrence, as they include asthenia, unconsciousness, decreased intestinal motility, electrocardiographic abnormalities (e.g., shortened QT interval), and acute kidney injury.^[1,2]^ Although the causes of hypercalcemia are varied, previous studies have reported that hyperparathyroidism and malignancy account for 80% to 90% of cases.^[1,3]^ The intake of vitamin D or granulomatous diseases, such as sarcoidosis, can also cause hypercalcemia. However, the incidence of hypercalcemia is reported to be quite low if only one of these factors is present.^[4–6]^

We herein report a case of hypercalcemia in a patient with a long-term history of sarcoidosis and a year-long use of cholecalciferol (vitamin D supplement), with the condition promptly restored by corticosteroid treatment.

## 2. Case

The patient was a 62-year-old man with a background of sarcoidosis. He was admitted to our hospital due to hypercalcemia and progressive decline of the kidney function. He had been suspected of having sarcoidosis due to symptoms such as uveitis and bilateral hilar lymphadenopathy about 30 years earlier, but no definitive diagnosis had been made. Uveitis was diagnosed 10 years before the admission and treated with oral prednisolone for about 9 months followed by ophthalmic prednisolone. He presented with erythema 5 years before his present admission, and based on the findings of a skin biopsy, he was diagnosed with sarcoidosis and started on topical steroids and tacrolimus hydrate. The erythema improved with the use of topical steroids, so the use of topical medication was stopped after 2 years.

About 6 months after the diagnosis of sarcoidosis, he was admitted to our hospital with alveolar bleeding and chylothorax leading to respiratory failure and was treated with methylprednisolone pulse therapy followed by oral prednisolone. During this treatment at our hospital, the levels of serum sarcoidosis markers of angiotensin-converting enzyme (ACE) and soluble interleukin-2 receptor (sIL-2R) were 7.8 U/L (normal range: 8.321.4 U/L) and 3550 U/mL (normal range: 154–474 U/mL) at the highest point, respectively, suggesting enhanced activity of sarcoidosis. However, no notable findings of hypercalcemia or kidney function decline were noted, with serum calcium and creatinine levels of 8.1 to 9.6 and 0.80 to 0.96 mg/dL, respectively.

While he was being followed up for sarcoidosis, he presented with fatigue and loss of appetite that started a few days before admission. At this time, we found that he had been taking an over-the-counter cholecalciferol supplement (100 μg/d) for about a year, which led to suspicion that his hypercalcemia was a result of vitamin D intoxication. Aside from the cholecalciferol medication, he had also been taking the following over-the-counter supplements: 3000 mg/d of vitamin C, 30 mg/d of zinc, 100 µg/d of selenium, and 400 mg/d of magnesium. Regarding prescribed medications, he had been prescribed 1350 mg/d of Quercus salicina extract and 25 µg/d of levothyroxine sodium hydrate. His medical history included appendicitis, glaucoma, hypothyroidism, and urolithiasis, with no family history of hypercalcemia.

**Figure 1.**
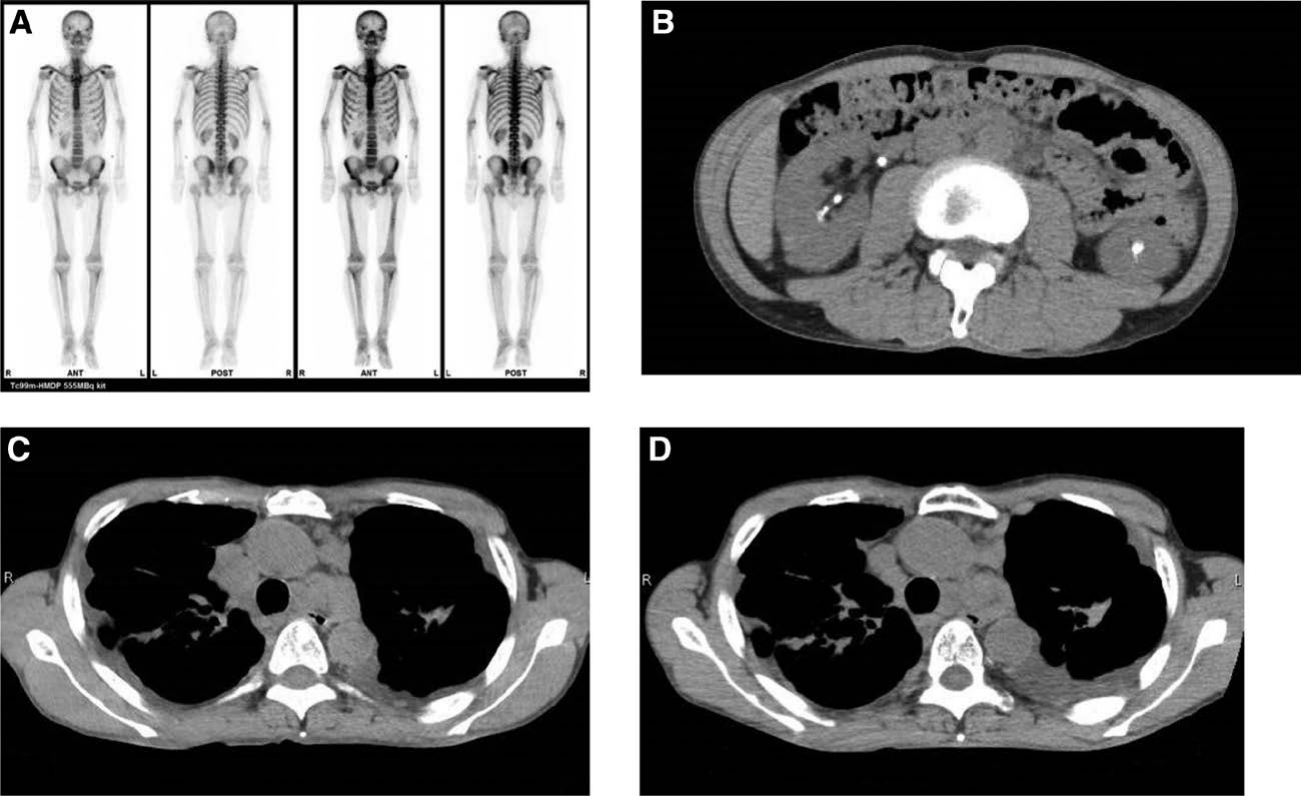
Imaging studies in the present case. (A) Bone scintigraphy showed no sign of bone malignancy. (B) Computed tomography of the abdomen taken at admission. Multiple nephrolithiasises were observed in both kidneys without evidence of hydronephrosis. (C and D) Computed tomography of the chest taken 6 months before admission (C) and at admission (D). Bilateral hilar lymphadenopathy shows no apparent size changes over the past 6 months.

A physical examination upon admission showed no abnormalities: height of 173 cm, body weight of 51 kg, and body mass index of 17. His vital signs were a blood pressure of 131/96 mm Hg, heart rate at 96 per minute, and body temperature of 37.1°C, also showing no abnormalities. The results of blood and urine analyses are shown in Table 1. The tests showed high levels of serum calcium at 13.2 mg/dL and creatinine at 2.28 mg/dL. The levels of intact parathyroid hormone and parathyroid hormone-related protein were 9 pg/dL and <1.0 pmol/L, respectively. The serum 1,25-dihydroxy-vitamin D_3_ (1,25-(OH)_2_ Vit.D_3_) level at the time of admission was high at 158 (normal range: 20–60) pg/mL, which is consistent with vitamin D-mediated hypercalcemia. An interferon-gamma release assay (T-Spot®) was negative. Sarcoidosis markers were moderately elevated: ACE of 21.8 U/L and sIL-2R of 3270 U/mL. We confirmed hypercalciuria in both casual and 24-hour urinalyses, with a urine calcium level of 28.6 mg/dL and 519.1 mg/d, respectively, and fractional excretion rate of Ca of 8.5% and 10.6%, respectively. Bone scintigraphy did not reveal any evidence of bone malignancy (Fig. 1A). Computed tomography (CT) showed multiple nephrolithiasises in both kidneys, although there was no evidence of hydronephrosis (Fig. 1B). We confirmed bilateral hilar lymphadenopathy without significant size changes over the past 6 months (Fig. 1C and D), suggesting that the activity of sarcoidosis was not significantly enhanced. We also detected no apparent malignancy in other organs.

**Table 1:** Laboratory findings at the time of admission.

Laboratory findings Serum		Reference range	Laboratory findings Serum		Reference range
WBC	7100/μL	3300–8600	Ca	13.2 mg/dL	8.8–10.1
Neutro%	75.6%	40.6–76.4	Mg	1.6 mg/dL	1.8–2.6
Lympho%	13.3%	16.5–49.5	P	2.9 mg/dL	2.7–4.6
Mono%	6.3%	2.0–10.0	i-PTH	9 pg/dL	10–65
Eosino%	3.7%	0.0–8.5	PTHrP	<1.0 pmol/L	<1.0 pmol/L
Baso%	1.1%	0.0–2.5	1.25 (OH)_2_Vit.D_3_	158 pg/mL	20–60
RBC	3.54 × 10^6^/μL	4.35–5.55 × 10^6^	κ/λ ratio	1.59	
Hemoglobin	11.4g/dL	13.7–16.8	M protein	(−)	
Hematocrit	32.3%	40.7–50.1	β2MG	5.6 mg/L	0.8–2.0
Platelets	236 × 10^3^/μL	158–348 × 10^3^	IgG	1850 mg/dL	861–1747
AST	14 U/L	13–30	IgA	299 mg/dL	93–393
ALT	10 U/L	10–42	IgM	54 mg/dL	33–183
LDH	116 U/L	124–222	PSA	2.42 ng/mL	0.0–4.0
ALP	76 U/L	106–322	T-SPOT	(−)	
T-Bil	0.5 mg/dL	0.4–1.5	sIL-2R	3200 U/mL	8.3–21.4
γ-GTP	27 U/L	13–64	ACE	24.8 U/L	157–474
Albumin	4 g/dL	4.1–5.1	PT-INR	0.91	
CRP	0.15 mg/dL	<0.14	APTT	28.9 s	24.0–36.0
CK	38 U/L	59–248	Fbg	354 mg/dL	150–400
UN	29 mg/dL	8.5–20	D-dimer	0.7 µg/mL	
Creatinine	2.27 mg/dL	0.65–1.07	pH	7.39	
eGFR	24.2 mL/min/1.73 m^2^		pCO_2_	53.5 Torr	
Na	139 mmol/L	138–145	HCO_3_-	31.6 mEq/L	
K	3.8 mmol/L	3.6–4.8	Ca^2+^	1.8 mmol/L	
Cl	101 mmol/L	101–108			
**Laboratory findings** **Casual urine**		Reference range	**Laboratory findings** **24-h urine**		Reference range
pH	6	13–30	Urine volume	2900 mL	
Specific gravity	1.009	1.005–1.030	Urine protein	0.1 g/d	
Protein	(+−)	(−)	U-UN	183 mg/dL	
Glucose	(−)	(−)	U-Cre	29.1 mg/dL	
Ketones	(−)	(−)	U-Na	104 mmol/L	
Bilirubin	(−)	(−)	U-K	13.6 mmol/L	
Urobilinogen	(+−)	(−)	U-Cl	101 mmol/L	
OCC	(2+)	(−)	U-Ca	519.1 mg/d	
RBC	50–99/HPF		FECa	10.6%	2%–4%
WBC	30–49/HPF		24hrCcr	31.5 mL/min	70–130 mL/min
uTP/Cr	0.28 g/gCr				
U-UN	323 mg/dL				
U-Cre	58.1 mg/dL				
U-Na	61 mmol/L				
U-K	17.3 mmol/L				
U-Cl	64 mmol/L				
U-Ca	28.6 mg/dL				
FECa	8.5%	2%–4%			
β2m/Cr	5682.7 µg/gCr				
NAG/Cr	17.2 IU/gCr				
BJP	(−)	(−)			

γ-GTP = gamma-glutamyl transpeptidase, ACE = angiotensin-converting-enzyme, ALP = alkaline phosphatase, ALT = alanine aminotransferase, APTT = activated partial thromboplastin time, AST = aspartate aminotransferase, Baso = basophil, CK = creatine kinase, CRP = C-reactive protein, eGFR = estimated glomerular filtration rate, Eosino = eosinophil, Fbg = fibrinogen, IgA = immunoglobulin A, IgG = immunoglobulin G, IgM = immunoglobulin M, i-PTH = intact parathyroid hormone, LDH = lactate dehydrogenase, Lympho = lymphocyte, Mono = monocyte, Neutro = neutrophil, PSA = prostate-specific antigen, PTHrP = parathyroid hormone-related protein, PT-INR = international normalized ratio of prothrombin time, RBC = red blood cell count, sIL-2R = soluble interleukin-2 receptor, T-Bil = total bilirubin, UN = urea nitrogen, WBC = white blood cell count.

**Figure 2.**
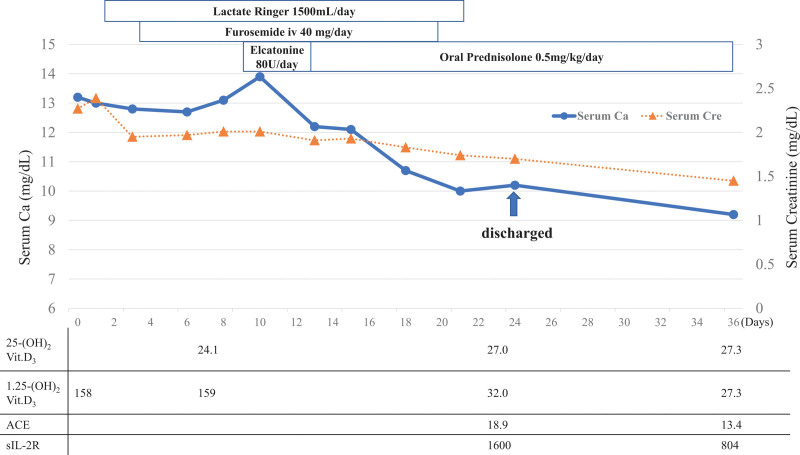
Clinical course of the present case. The solid-line circle and dotted-line triangle indicate the serum calcium and creatinine levels, respectively. ACE = angiotensin-converting enzyme, sIL-2R = soluble interleukin-2 receptor.

After admission, the intake of cholecalciferol supplement was discontinued, and intravenous fluid treatment of lactate ringer at 1500 mL/d with 40 mg/d of furosemide were started (Fig. 2). On the 7th day of admission, the serum calcium was 13.9 mg/dL, showing worsening. We therefore added 80 U/d of elcatonin for 3 days, but the serum calcium level was unaffected. On the 7th day of admission, the serum 1,25-(OH)_2_ Vit.D_3_ level remained high at 159 pg/mL. In contrast, the serum 25-hydroxy vitamin D_3_ (25-OH Vit.D_3_) level was low at 24.1 (normal range: >30) ng/mL. These results indicated that the conversion from 25-OH Vit.D_3_ to 1,25-(OH)_2_ Vit.D_3_ was significantly increased, given he had taken adequate doses of cholecalciferol supplement over the past year. Considering his history of sarcoidosis and moderate elevation of sarcoidosis markers, we posited that the 1-α-hydroxylase expression or activity was enhanced, possibly due to production by sarcoidosis lesions. Thus, oral prednisone 0.5 mg/kg/d to inhibit the activity of sarcoidosis was administered from the 13th day. A week later, serum calcium and creatinine levels dropped to 10.1 and 1.74 mg/dL, respectively.

He was discharged on the 23rd day without any evidence of adverse events induced by corticosteroid treatment. The 1.25-(OH)_2_ Vit.D_3_ and 25-OH Vit.D_3_ levels at the time of discharge were 32.0 pg/dL and 27.0 ng/dL, respectively.

## 3. Discussion

We experienced a case of hypercalcemia and acute kidney injury in a patient with sarcoidosis who had taken an over-the-counter cholecalciferol supplement for over 1 year. The concomitant measurement of 25-OH Vit.D_3_ and 1,25-(OH)_2_ Vit.D_3_ levels played a crucial role in the diagnosis of the case. While resistant to standard treatments against hypercalcemia, such as intravenous fluid therapy, furosemide, and calcitonin, it was quite sensitive to corticosteroid treatment.

Hypercalcemia is common disorder induced by various causes, with previous studies reporting that hyperparathyroidism and malignancy account for over 80% of cases.^[1,3]^ In contrast, the prevalence of hypercalcemia induced by sarcoidosis alone is relatively rare, occurring in approximately 10% of sarcoidosis patients.^[4,5]^ In addition, an interventional study by McCullough et al^6]^ showed that the average serum calcium levels in subjects who took over-the-counter cholecalciferol supplement (250 μg/d) for over 4 years were 9.4 to 10 mg/dL, suggesting that the long-term intake of cholecalciferol supplement does not always cause hypercalcemia.

Vitamin D plays versatile roles in our system, the main one being regulating the serum calcium level. In addition, vitamin D_3_ has an influence over our immune system. For example, 1,25-(OH)_2_ Vit.D_3_ enhances macrophage differentiation, leading to the formation of epithelioid cell granuloma.^[7]^ This epithelioid cell granuloma produces 1-α-hydroxylase, which enhances the conversion of 25-OH Vit.D_3_ to 1,25-(OH)_2_ Vit.D_3_.^[8]^ In addition, vitamin D_3_ is a fat-soluble vitamin, meaning that its excess intake accumulates mainly in our adipose tissue as cholecalciferol (vitamin D_3_).^[9]^ Vitamin D_3_ is converted into 25-OH Vit.D_3_ by 25-hydroxylase in our liver, and this 25-OH Vit.D_3_ is the major circulating form of vitamin D in our system.^[10]^

Although the vitamin D_3_ supplement in our patient was discontinued at the time of his admission, the serum 1,25-(OH)_2_ Vit.D_3_ level remained high, and the serum 25-OH Vit.D_3_ level remained low. With imaging test results ruling out the possibility of malignancy, the vitamin D data led to the diagnosis that the increased expression or activation of 1-α-hydroxylase caused by the enhanced activity of sarcoidosis had caused the hypercalcemia in this case. The fact that the hypercalcemia was resistant to standard treatment but sensitive to corticosteroid treatment also supported the notion that the sarcoidosis played a role in the development of hypercalcemia. In addition, the improvement of sarcoidosis markers, such as sIL-2R and ACE, further supported this hypothesis.

Inui et al^11]^ reported that there were significant correlations between the 1-α-hydroxylase mRNA levels in bronchoalveolar lavage samples, the percentage of alveolar lymphocytes, and the serum ACE level, suggesting that increased disease activity of sarcoidosis leads to increased 1-α-hydroxylase levels. However, there was no evidence of hypercalcemia when the patient experienced respiratory failure due to sarcoidosis 4 years earlier, which we presumed that was the highest disease activity of sarcoidosis. This fact suggested that the hypercalcemia was the result of not only a high disease activity of sarcoidosis but also the intake of cholecalciferol supplement. Unfortunately, the serum 1,25-(OH)_2_ Vit.D_3_ and 25-OH Vit.D_3_ levels were not measured when he suffered from respiratory failure. We also concluded that the discontinuation of cholecalciferol supplement was not sufficient, and corticosteroid therapy was needed to improve his hypercalcemia, as vitamin D_3_ is fat-soluble, and its excess accumulation might remain in his body for a long period of time.

Our case suggested that physicians should keep a close eye on patients’ medications, including over-the-counter supplements, especially in those with granulomatous diseases. Regular measurements of the serum calcium level and kidney function are needed to detect abnormal calcium metabolism in patients with granulomatous diseases. Furthermore, the concomitant measurement of 25-OH Vit.D_3_ and 1,25-(OH)_2_ Vit.D_3_ levels is useful for determining the cause of hypercalcemia.

## Author contributions

MK and KH drafted the manuscript; MK, KH, AS, KO, and RK were involved in patient management; MI and TY supervised the report; all authors approved the final manuscript and agreed to submit this work to this journal.
